# miR-155 and miR-92 levels in ALL, post-transplant aGVHD, and CMV: possible new treatment options

**DOI:** 10.1186/s43046-023-00174-3

**Published:** 2023-06-19

**Authors:** Mahdiyar Iravani Saadi, Mohsen Nikandish, Zahra Ghahramani, Fatemeh Mardani Valandani, Maryam Ahmadyan, Fakhroddin Hosseini, Zahra Rahimian, Heeva Jalali, Fataneh Tavasolian, Ehsan Nabi Abdolyousefi, Nadiya Kheradmand, Mani Ramzi

**Affiliations:** 1 Hematology Research Center, Shiraz University of Medical Sciences, Mohammad Rasul Allah Research Tower, Opposite the Education School, Khalili Ave, Shiraz, Fars Iran; 2 Hematology, Oncology and Bone Marrow Transplantation Department, Shiraz University of Medical Sciences, Namazi Sq., Zand St., Shiraz, Iran; 3 Department of Animal Science, Faculty of Agriculture, University of Kurdistan, Pasdaran Blvd, Sanandaj, Kurdistan Iran

**Keywords:** miR-155, miR-92, Acute lymphoblastic leukemia, CMV, aGVHD, MicroRNA, Post-transplant

## Abstract

**Background:**

Acute lymphoblastic leukemia (ALL) is a malignancy that leads to altered blast cell proliferation, survival, and maturation and eventually to the lethal accumulation of leukemic cells. Recently, dysregulated expression of various micro-RNAs (miRNAs) has been reported in hematologic malignancies, especially ALL. Cytomegalovirus infection can induce ALL in otherwise healthy individuals, so a more detailed evaluation of its role in ALL-endemic areas like Iran is required.

**Methods:**

In this cross-sectional study, 70 newly diagnosed adults with ALL were recruited. The expression level of microRNA-155(miR-155) and microRNA-92(miR-92) was evaluated by real-time SYBR Green PCR. The correlations between the miRNAs mentioned above and the severity of disease, CMV infection, and acute graft vs. host disease after hematopoietic stem cell transplantation (HSCT) were assessed. B cell and T cell ALL distinction in the level of miRNAs was provided.

**Results:**

After the statistical analysis, our results indicated a marked increase in the expression of miR-155 and miR-92 in ALL patients vs. healthy controls (*P = 0.002–*P = 0.03, respectively). Also, it was shown that the expression of miR-155 and miR-92 was higher in T cell ALL compared to B cell ALL (*P* = 0.01–*P* = 0.004, respectively), CMV seropositivity, and aGVHD.

**Conclusion:**

Our study suggests that the plasma signature of microRNA expression may act as a powerful marker for diagnosis and prognosis, providing knowledge outside cytogenetics. Elevation of miR-155 in plasma can be a beneficial therapeutic target for ALL patients, with consideration of higher plasma levels of miR-92 and miR-155 in CMV + and post-HSCT aGVHD patients.

## Introduction

Acute lymphoblastic leukemia (ALL) is a malignancy of lymphoid progenitor cells. Occurring 80% of the time in children, it still constitutes a catastrophic disease when it comes to adults [1]. ALL is classified as B and T lymphoblastic leukemia (T-ALL, B-ALL) [2]. In adults, 75% of cases develop from precursors of the B cell lineage, with the remainder of cases consisting of malignant T cell precursors [1]. ALL can be cured in 90% of children, whereas only 40% of adult patients respond to treatment, possibly due to chromosomal abnormality and insensitivity to treatment. The hallmarks of ALL are chromosomal abnormalities and genetic alterations impacting the differentiation and proliferation of lymphoid precursor cells [1, 3]. Many ALL subtypes are characterized by constellations of structural rearrangements, submicroscopic DNA copy number alterations, and sequence mutations, several of which have clear implications for risk stratification and targeted therapeutic intervention [4]. Recently, an increasing number of studies showed that the microRNA (miRNA) expression profiles in acute leukemia have cooperative interactions in the development of leukemia. Therefore, the miRNA expression profile can be used as biomarkers in diagnosis, differential diagnosis, prognosis, and therapy of hematologic cancers [5]. In developed countries, the overall survival of patients with ALL has increased to more than 80%; however, those children cured of ALL still show a significant risk of short and long-term complications as a consequence of their treatment. Accordingly, there is a need not only to develop new methods of diagnosis and prognosis but also to provide patients with less toxic therapies [6].

### miRNAs

miRNAs are small, non-coding 18–24 bp dsRNAs that can post-transcriptionally regulate the majority of protein-coding as well as non-coding genes in different cellular processes via direct mRNA degradation or translational blockade [7]. MiRNAs are expressed differentially in distinct stages of lymphopoiesis and influence the direction of lymphoid precursor maturation. Hence, there is an aberrant expression of miRNAs involved in malignant lymphopoiesis, and these aberrations can be used as signatures of ALL with different subtypes [5]. miRNAs are known to have both tumor-suppressive and oncogenic functions in leukemia [8].

MicroRNA-155 (miR-155), an oncogenic miRNA, participates in multiple biological pathways, including hematopoiesis, inflammatory response, and immune system. It is located on a non-coding RNA exon transcribed from the B cell integration cluster found on chromosome 21 [9]. It is found in both lymphoid and myeloid cells but at different levels, based on the type of cell. miR-155 facilitates the growth of germinal centers and plasma B cells [10] and holds one of the highest miRNA-target interactions between leukemia-associated genes and miRNAs associated with ≥ 4 leukemia types [8].

The polycistronic microRNA cluster microRNA-17–92 (miR-17–92) encodes miR-17, miR-18a, miR-19a, miR-20a, miR-19b-1, and miR-92. In multiple cancers, such as acute myeloid leukemia, malignant lymphoma, and lung cancer, the human microRNA cluster miR-17–92 is overexpressed [11]. Some reports also propose miR-92 dysregulation in hematopoietic and solid cancers [11–13]. miR-17‐92 is also one of the miRNAs regulating RUNX1, a transcription factor in hematopoiesis expression [8]. However, downregulation of miR-92 in human plasma has been considered a potential indicator for patients with acute leukemia [14].

### Acute graft-vs-host disease

Acute GVHD (AGVHD) remains a common complication of allogeneic hematopoietic stem cell transplantation (HSCT), with a significant impact on early morbidity and mortality [15]. GVHD occurs when transplanted donor T lymphocytes react to foreign host cells. It causes a wide variety of host tissue injuries [16]. AGVHD severity is usually graded (grades 0–IV) by the pattern of organ involvement using the classic Glucksberg–Seattle criteria [17]. According to the Glucksberg grade index, GVHD is categorized into four grades:grade I—stage 1 or 2 skin involvement; no liver or gut involvement; grade II—stage 1 to 3 skin involvement; grade 1 liver or gut involvement; grade III—stage 2 or 3 skin, liver, or gut involvement; grade IV—stage 1 to 4 skin involvement; stage 2 to 4 liver or gut involvement [18].

### Cytomegalovirus (CMV)

CMV is prevalent globally, especially in Iran, where the baseline is estimated 91.8% of the population [19, 20]. Although a benign infectious agent in the healthy, CMV is a notorious driver of morbidity and mortality in hematological patients with failed immunocompetence [21]. Cytomegalovirus infection is one of the most significant viral complications of allogeneic HSCT [21]. Patients are at a higher risk of developing aGVHD during CMV replication [22]. Congenital CMV infection was recently identified as a risk factor for childhood acute lymphocytic leukemia by detecting the presence of CMV sequences in neonatal blood spots [23]. Recent evidence supports the role of cytomegalovirus (CMV) in the development of childhood ALL. The underlying mechanism and CMV’s role in the leukemic cell phenotype are unknown, but CMV typically interacts with the host immune system allowing the virus to survive in a latent state; it may be that this immune dysregulation affects the risk of ALL [24]. CMV reactivation was reported to reduce the risk of relapse after allogenic HSCT for pediatric acute leukemia. However, it does not attribute to a survival benefit due to opportunistic infections after grades II to IV aGVHD and CMV reactivation [25]. Still, the direct relationship between CMV, miR-92, and miR-155 has not been determined.

In this study, we have focused on the correlations between miR-155 and miR-92 and ALL, their link with thedevelopment of aGVHD after HSCT, and their relation to CMV.

## Patients and methods

### Inclusion criteria

In this cross-sectional study, 70 newly diagnosed adults (completely random) with acute lymphoblastic leukemia were admitted to a referral hospital for hematological malignancies, and 70 normal controls from 2016 to 2018 were recruited. Acute lymphoblastic leukemia was diagnosed by an expert oncologist using morphology, cytochemistry, and immunophenotyping. Clinical and laboratory data were also collected, including WHO subclass, complete blood count, blast percentage, and hemoglobin (Hb) level. All patients received standard induction chemotherapy consisting of cycle A and cycle B. Cycle A is as follows: cyclophosphamide 300 mg/m^2^ every 12 h, with 6 doses, days 1, 2, and 3; mesna iv 1200 mg/m^2^/day continuous infusion, days 1, 2, and 3; vincristine iv 1.4 mg/m^2^/day, 4th and 11th day; doxorubicin 50 mg/m^2^/day, 4th day; dexamethasone 40 mg/day, days 1–4 and 11–14. Cycle B is as follows: methotrexate 1 g/m^2^ continuous infusion, day 1; leucovorin 15 mg every 6 h for 8 doses, starting 12 h after the end of methotrexate infusion; cytarabine 3 g/m^2^ every 12 h, for 4 doses, days 2 and 3 [26]. A total of 37 patients received HSCT from the related HLA-matched donors. aGvHD was classified according to the classic Glucksberg–Seattle criteria. Sixteen patients developed aGvHD, 7 patients had low grade (grade I + II) aGvHD, and 9 cases developed high grade (grade III + IV) aGvHD. All procedures were under the Helsinki protocol of 1975 and its later amendment. It was also approved by the local ethics committee of Shiraz University of Medical Sciences (Shiraz, Iran) (ethics committee code #1396–01-01–14,570).

### Cytogenetic analysis

Patients’ karyotype was analyzed by standard G-banding technique [27]. Chromosomal abnormalities were tested by reverse transcriptase polymerase chain reaction (RT-PCR) for BCR/ABL, TEL/AML1, and E2A/PBX1. Patients who were negative for these chromosomal abnormalities were considered CN-ALL. Among the 70 ALL patients, 47 had normal cytogenetic, and 23 had abnormal karyotypes.

### Sample collection and ribonucleic acid isolation

Five-milliliter peripheral blood was collected in ethylenediaminetetraacetic acid (EDTA)-containing tubes from each patient at the time of diagnosis prior to chemotherapy treatment and also from healthy individuals. The peripheral blood mononuclear cells were isolated from each patient and controls using Ficoll-Hypaque density gradient centrifugation. Total RNA was extracted by TRIZOL reagent (Invitrogen) according to the manufacturer’s instructions, as previously described briefly [28–30].

### Quantification of the miR-155 and miR-92mRNAs expression level by SYBR Green Real-time PCR

For the quantitative analysis of miR-181a and miR-181b mRNA expression level, the SYBR Green Real-Time PCR method was performed using SYBR Premix Ex TaqTM II (TliRNaseH Plus) (Takara, Japan). Primers were designed specific for each miRNA in an iQ5 thermocycler (BioRad Laboratories, USA) according to the manufacturer’s instructions, as previously described briefly [28–30]. GAPDH was used as the internal control in expression studies. Primers designed for each miRNA in an iQ5 thermocycler (BioRad Laboratories, USA) using the Primer-BLAST are listed in Table 1.

**Table 1:** The primer sequences for qRT-PCR and PCR condition used for the miR-181a and miR-181b and GAPDH gene

Gene	Primer sequences	Thermocycling condition
GAPDH	Forward GGACTCATGACCACAGTCCA Reverse CCAGTAGAGGCAGGGATGAT	95 °C/2 min, 40 cycles of 95 °C/30 s, 57.5 °C/20 s, and 70 °C/30 s
MIR-92a	Forward GTGCAGGGTCCGAGGT Reverse GTGCAGGGTCCGAGGT	94 °C/2 min, 40 cycles of 94 °C/30 s, 57 °C/20 s, and 70 °C/30 s
MIR-155	Forward GCTACTCCTACATATTAGCA Reverse GTGCAGGGTCCGAGGT	95 °C/2 min, 40 cycles of 95 °C/30 s, 58 °C/20 s, and 70 °C/30 s

### CMV antigenemia assay

CMV antigenemia testing was carried out using EDTA whole blood, as defined in the box inserts of the CMV Brite Turbo kit (IQ Products, Groningen, the Netherlands). Cyto-centrifuged preparations (Cytospine3, Shandon Scientific, Cheshire, England) were used [31].

### Statistical analysis

Data were analyzed by the SPSS software, version 18. The differences in the mean expression level of miR-155 and miR-92 between patients and controls, as well as WHO subtypes, were compared via independent *t*-test. The association between the mean expression of the miR-155 and miR-92 and laboratory data were analyzed by Pearson correlation test.

## Results

Of the 70 newly diagnosed ALL patients, 46 (65.7%) were male. The mean age of ALL patients was 42 ± 1.3 with a range of 15‐65 years. Patients’ demographic data included mean white blood cell (WBC) counts (50,032 ± 9,881), platelet count (51,651 ± 6541), Hb level (g/dL) (9.2 ± 0.54), and lactate dehydrogenase (LDH) level (U/L) (1352 ± 189).

### Aberrant miR-155 and miR-92 expression in ALL patients

**Fig. 1:**
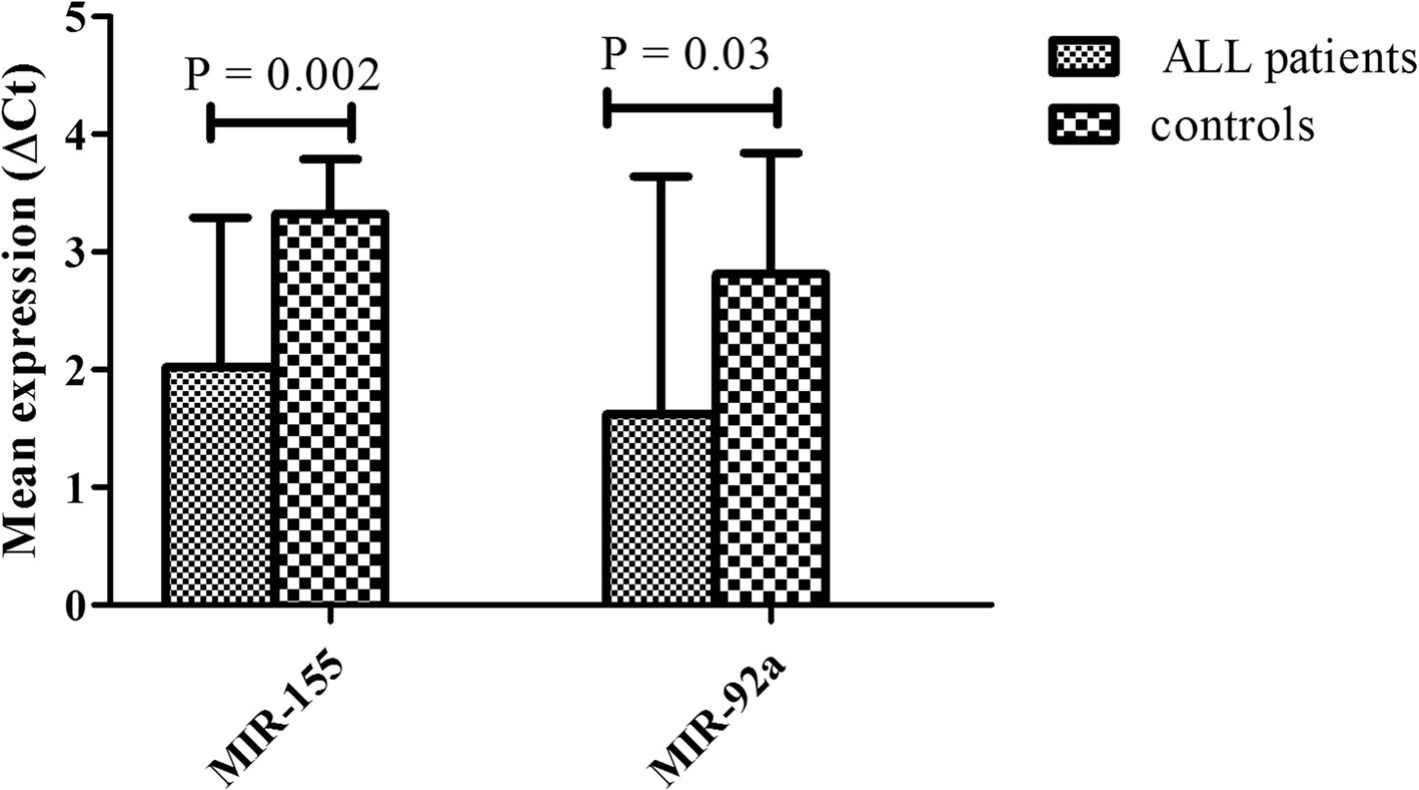
miR-155 and miR-92 expression in ALL patients

The mRNA expression of miR-155 and miR-92was compared between patients and controls (Fig. 1). After the statistical analysis, our results revealed that the expression of miR-155 and miR-92 was significantly higher (5.6 fold, 4.2) in ALL patients vs. healthy controls (2.1 ± 0.65 vs. 3.4 ± 1.5, *P = 0.002–1.7 ± 0.16 vs. 2.8 ± 0.64, *P = 0.03, respectively).

### miR-155 and miR-92expression according to B cell or T cell ALL

In this study, we evaluated the expression of miR-155 and miR-92 in ALL patients, according to B cell or T cell ALL. Our results revealed that the expression of miR-155 and miR-92 was higher in T-ALL than patient’s B-ALL (− 1.7 ± 0.24 vs. 2.5 ± 1.1; *P* = 0.01, 1.6 ± 0.21 vs. 4.2 ± 0.87; *P* = 0.004).

### miR-155 and miR-92 expression according to cytogenetic status

The expression level of miR-155 and miR-92 was compared within ALL patients based on their cytogenetic abnormalities. Cytogenetic findings in ALL patients and their details are shown in Table 2. Among 70 ALL patients, 47 had normal cytogenetics, and 23 had abnormal karyotypes. Our results showed that the expression level of both miR-155 and miR-92 had significantly increased in cytogenetic abnormalities cytogenetic aberrations (BCR/ABL t(9;22) (*P* = 0.03, *P* = 0.01, respectively)). Also, our results showed that the expression level of miR-155 and miR-92 did not differ between patients with different cytogenetic aberrations (TEL/AML1, E2A/PBX1) (*P* > 0.05).

**Table 2:** Acute lymphoblastic leukemia with recurrent cytogenetic abnormalities

**Cytogenetic abnormalities**	No. of patients (%)
t (9;22) BCR/ABL	13 (18.5%)
t (12;21) TEL/AML1	6 (8.5%)
t (1;19) E2A/PBX1	4 (5.7%)

### miR-155 and miR-92 expression in HSCT patients and development of the acute GVHD

The mean expression of miR-155 and miR-92 was compared between patients with and without aGVHD. Our results showed that miR-155 gene expression levels had significantly increased in patients who developed aGVHD compared to those without aGVHD (1.23 ± 1.6 vs. 4.5 ± 2.3; *P* = 0.03). Also, our results revealed that the expression of miR-92was not significantly higher in patients who had developed aGVHD in comparison with those without aGVHD (2.4 ± 1.3 vs. 4.1 ± 1.6; *P* = 0.2). In addition, miR-155 and miR-92 were overexpressed in HSCT patients with high-grade aGVHD (grades III–IV) compared to those patients who developed low-grade (grades 0–II) aGVHD, but the difference was not statistically significant (− 1.4 ± 3.2 vs. 3.1 ± 5.2; *P* = 0.6, for miR-155) (1.6 ± 4.2 vs. 2.5 ± 6.1; *P* = 0.3, for miR-92).

### Association of MiR-92a and MiR-155 expression with CMV infection in ALL patients

**Fig. 2:**
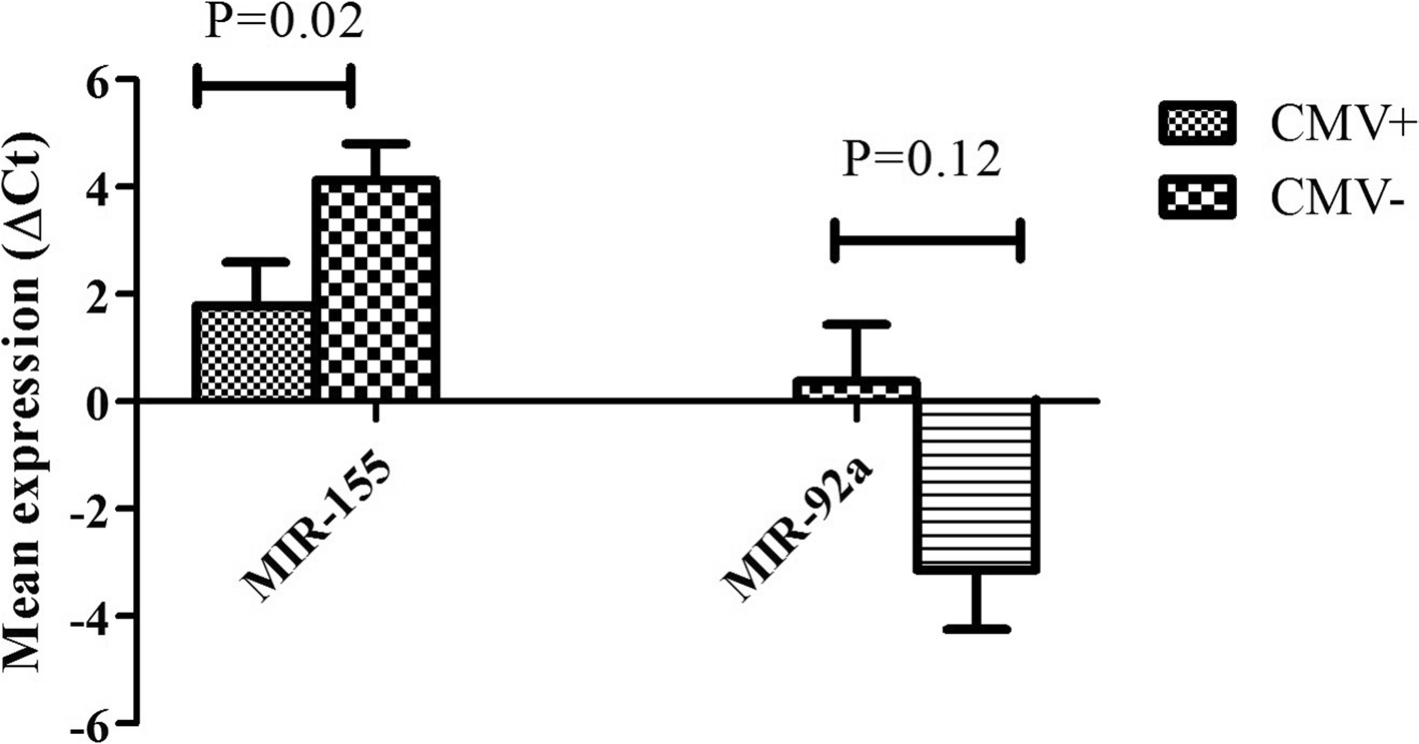
miR-92a and miR-155 in CMV infection in ALL patients

CMV was detected in 11 of 70 (15.7%) patients. The mean expression of MiR-92a and MiR-155 was compared in patients according to the CMV infection status (Fig. 2). Our results demonstrated that MiR-155 expression levels were increased significantly in CMV^+^ patients in comparison to CMV^−^ patients(*P* = 0.02). Albeit, the miR-92a expression level was decreased in CMV^+^ patients compared to CMV^−^ patients (*P* = 0.12).

## Discussion

The expression profiling of miRNAs in ALL could soon be used to classify the disease, establish specific diagnoses, and offer prognostic values [21].

Our results revealed that the miR-155 and miR-92 expressions were significantly higher (5.6 fold, 4.2) in ALL patients vs. healthy controls. Studies indicate that miR-155 deregulation is related to several types of cancer, respiratory disorders, and viral infections and, more specifically in ALL, is upregulated [32]. A significant correlation has been reported between high miR-155 levels and high blast numbers (> 25%), unfavorable cytogenetic abnormality, total WBC, and a higher relapse rate. The overexpression of miR-155, therefore, correlates with poor prognosis in pediatric ALL due to a decrease in response to therapy and an increase in relapse. Furthermore, after therapy, miR-155 expression levels were significantly decreased tenfold, again suggesting potential use as a biomarker of therapeutic response in childhood ALL [6, 33]. As for miR-92, its plasma levels were found to be elevated in ALL cases at the time of diagnosis (2.51 and 2.19 folds) and relapse (2.1 and 1.61 folds) than that of patients with remitted ALL [34].

Xie et al. found that miR-155 was significantly upregulated in GVHD patients after allogeneic peripheral blood stem cell transplantation and showed a correlation between the level of miR-155 and the severity of aGVHD. Blockage of miR-155 expression leads to decreased GVHD sensitivity and prolonged survival [35]. Our study showed that miR-155 gene expression levels were significantly increased in patients who had developed acute GVHD compared to those who did not. Also, the obtained results revealed that the expression of miR-92 was not remarkably higher in patients who had developed acute GVHD than in those who did not. In addition, miR-155 and miR-92 were overexpressed in HSCT patients with high-grade aGVHD (grades III–IV) compared to those who had developed lower grades (grades I–II) of the disease. However, it is not yet elucidated whether the downregulation of miR-92 is an indicator for patients with acute leukemia [14] or its overexpression [11–13]. Although the difference was not statistically significant, in the future, anti-microRNA can be used for the treatment of ALL patients and also reduce GVHD after HSCT in the mentioned patients. Congenital cytomegalovirus (CMV) infection was recently identified as a risk factor for childhood acute lymphocytic leukemia. Patients are at a significantly increased risk of developing aGVHD during CMV replication [22]. CMV reactivation may reduce the risk of relapse after HSCT for pediatric acute leukemia. As shown in Fig. 2, we indicated that the expression of miR-155 was elevated notably in CMV + patients compared to CMV‐ones, while miR-92 was decreased in CMV seropositivity, whereas 6 demonstrated that the expression of miR-155 decreased significantly with a coinciding increase in miR-92 in CMV^+^ patients [36]. The mentioned dis-concordance can be attributed to possible complex pathways.

Our study showed that miR-155 and miR-92 expression in T-ALL was more dominant than in B-ALL. During CD8 T cell differentiation, miR-155 increases, while a decrease in miR-92 coincides [37]. As for a wide range of B cells, an increase in miR-155 expression [38] is observed with an indirect association with CMV seropositivity via TNF-α [39]. Overexpression of the miR-17–92 polycistron is strongly associated with B cell lymphomagenesis [40, 41].

The majority of adults with ALL will ultimately relapse. In addition, up to 20% will have primary resistant diseases. The prognosis of adults with relapsed or refractory ALL is generally poor. Median survival is less than 1 year, and less than one quarter of patients survive up to 3 years. Given that resistance and relapse are the primary obstacles in treating ALL, developing additional and more personalized therapy methods is of great significance. Hence, it seems that we might be able to use anti-miRNA as an adjuvant to chemotherapy to treat refractory and relapse ALL, similar to AML [42, 43]. miRNAs have been used as a diagnostic factor to distinguish insensitivity to treatment in ALL patients[5] and to overcome that. They also have the potential to be applied as an adjuvant or as an alternative to conventional therapies for ALL [44]. Reversal of the expression of these miRNAs and anti-miR applications may have the potential for clinical use of adjuvant to or as an alternative to conventional therapies for childhood acute lymphoblastic leukemia [5, 45].

## Conclusion

Our study suggests that the plasma signature of microRNA expression may act as a powerful marker for diagnosis and prognosis, providing knowledge outside cytogenetics. Elevation of miR-155 in plasma can be a beneficial therapeutic target for ALL patients, with consideration of higher plasma levels of miR-92 and miR-155 in CMV + and post-HSCT aGVHD patients.

## Data Availability

The datasets used and/or analyzed during the current study are available from the corresponding author upon reasonable request.

## Abbreviations

ALL: Acute lymphoblastic leukemia
miRNA: MicroRNA
CMV: Cytomegalovirus
aGvHD: Acute graft versus host disease
HSCT: Hematopoietic stem cell transplant
WHO: World Health Organization
Hb: Hemoglobin
RT-PCR: Reverse transcriptase polymerase chain reaction
EDTA: Ethylenediaminetetraacetic acid
PBMCs: Peripheral blood mononuclear cells
LDH: Lactate dehydrogenase
WBC: White blood cell
